# Replenishment of TCA cycle intermediates provides photoreceptor resilience against neurodegeneration during progression of retinitis pigmentosa

**DOI:** 10.1172/jci.insight.150898

**Published:** 2021-09-08

**Authors:** Ashley A. Rowe, Pinkal D. Patel, Ruth Gordillo, Katherine J. Wert

**Affiliations:** 1 Department of Ophthalmology,; 2 Touchstone Diabetes Center, Department of Internal Medicine,; 3 Department of Molecular Biology, and; 4 Hamon Center for Regenerative Science and Medicine, University of Texas (UT) Southwestern Medical Center, Dallas, Texas, USA.

**Keywords:** Metabolism, Ophthalmology, Cell stress, Glucose metabolism, Neurodegeneration

## Abstract

The metabolic environment is important for neuronal cells, such as photoreceptors. When photoreceptors undergo degeneration, as occurs during retinitis pigmentosa (RP), patients have progressive loss of vision that proceeds to full blindness. Currently, there are no available treatments for the majority of RP diseases. We performed metabolic profiling of the neural retina in a preclinical model of RP and found that TCA cycle intermediates were reduced during disease. We then determined that (a) promoting citrate production within the TCA cycle in retinal neurons during disease progression protected the photoreceptors from cell death and prolonged visual function, (b) supplementation with single metabolites within the TCA cycle provided this therapeutic effect in vivo over time, and (c) this therapeutic effect was not specific to a particular genetic mutation but had broad applicability for patients with RP and other retinal degenerative diseases. Overall, targeting TCA cycle activity in the neural retina promoted photoreceptor survival and visual function during neurodegenerative disease.

## Graphical abstract

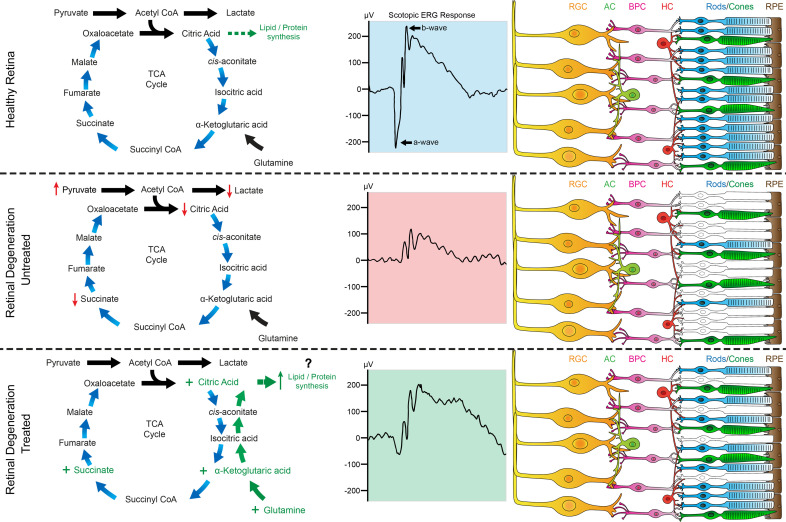

## Introduction

Postmitotic neuronal cell populations are particularly sensitive to the effects of aging, as they are unable to undergo mitosis and regenerate (1). The death of neuronal cells leads to a loss of quality of life, and often severe neurological impairment, for patients suffering from these conditions. Recently, studies have shown that mitochondrial dysfunction, impaired glucose metabolism, and abnormal aerobic glycolysis play key roles in age-related neurodegenerative diseases, such as Parkinson’s disease, Huntington’s disease, age-related macular dystrophy, and Alzheimer’s disease (2–5). These studies suggest that it is critical for the metabolic environment to be properly maintained in neuronal cells in order to preserve cellular health.

One of the most metabolically active tissues in the human body is the retina (6–8). The retina is comprised of a highly organized network of neuronal cells interconnected by synapses. The primary light-sensing neurons of the retina are the photoreceptors, responsible for excitation to the inner neuronal cell layers of the retina for visual processing of optical images by the brain. Although mutations in more than 271 genes have now been linked to retinal degenerative disease — characterized by the death of the photoreceptor neurons — there are currently no available treatment options to prolong vision and halt cell death for the majority of these diseases (9). Because retinal degenerative diseases have this high genetic heterogeneity, mutations in different genes can lead to the same clinical manifestation of disease. This suggests that there are common mechanisms underlying photoreceptor degeneration unrelated to the causative genetic mutation. Targeting these common cellular pathways, such as cellular metabolism, that are affected during neurodegenerative disease progression can lead to cost-effective therapeutic options for patients that do not rely on treating the individual genetic mutation.

Various research studies have begun testing gene therapy vectors that target photoreceptor cell metabolism. Previously, we found that silencing tuberin, an inhibitor of the mTOR pathway, prolonged photoreceptor cell survival in a mouse model of retinitis pigmentosa (RP), a group of inherited retinal degenerative diseases that leads to the death of the rod photoreceptor neurons, followed by secondary death of the cone cells (10, 11). Since then, additional studies have found that enhancing glycolytic metabolism, mTOR activity, or glucose transport in the photoreceptor neurons can slow degeneration in models of retinal disease (12–14). Each of these studies points to an important role for reprogramming to anabolism in the photoreceptor cell, particularly through the activation of the mTOR signaling pathway. However, a recent study found that mTOR activation in the cone photoreceptors led to age-related macular degeneration in the mouse (15). This suggests that there are likely different metabolic demands for the photoreceptor neurons depending on whether they are in a healthy or a diseased environment. Thus, there is a need to study the precise metabolic requirements that will protect neural retinal cells during disease progression.

Current research advances in understanding the metabolic flux between the neural retina and retinal pigment epithelium (RPE) have provided insight into a metabolic “ecosystem” in which the cells provide nutrients to support one another (16–20). However, these studies have mainly examined metabolism and metabolic flux in vitro in explanted retinal tissue, and there is limited in vivo information for metabolism and metabolic alterations during neurodegenerative disease progression. To examine metabolic requirements in vivo during disease, we previously screened retina and vitreous samples from a preclinical mouse model of autosomal recessive (ar) RP (arRP), carrying a missense mutation in the α subunit of phosphodiesterase 6 (*Pde6*α), to look for proteomic changes during disease progression (21). In our proteomics analysis, we found that cellular metabolic pathways were highly disrupted at the onset of photoreceptor degeneration (postnatal day 15 [P15] in the mouse), and we validated these findings with human vitreous biopsies from patients with RP caused by mutations in *PDE6A* (21). We then performed an in vivo screen in our arRP mouse model to gain insight into the metabolic pathways critical to the photoreceptor neurons. We provided dietary supplementation of individual metabolites targeting different cellular energy pathways and tested the mice for increased photoreceptor survival and visual response. Although many metabolites failed to have a significant effect on disease progression, one metabolite, α-ketoglutarate (α-KG), prolonged visual responses and photoreceptor survival for 1 month of age.

In our current study, we examined the potential mechanism by which α-KG supplementation provides resilience to the photoreceptor neurons against cell death during neurodegenerative disease progression. We hypothesized that since the TCA cycle intermediates feed into biosynthetic pathways that produce lipids, proteins, and nucleic acids to support the cell, the reductive carboxylation of glutamine-derived α-KG to isocitrate can assist the cell in maintaining pools of biosynthetic precursors to preserve photoreceptor cell health during disease progression (22, 23). We examined the effect of α-KG supplementation on precise TCA cycle metabolites using mass spectrometry, as well as testing for the duration of treatment effect in our preclinical arRP model. We found that α-KG supplementation enhanced citrate levels in the neural retina at 1 month of age. We then treated our arRP preclinical mouse model with citrate, and found that citrate supplementation improved photoreceptor resilience against cell death during RP disease progression and prolonged visual function beyond that of α-KG supplementation. Glutamine treatment followed α-KG supplementation effects, whereas replenishing another TCA cycle intermediate, succinate, provided enhanced resilience to the photoreceptor neurons against cell death during RP disease, similar to that of citrate supplementation. As metabolite supplementation does not correct the causative genetic mutation leading to RP disease progression, we hypothesized that the ability of α-KG and citrate to provide resilience against cell death to the photoreceptor neurons is not specific to mutations within PDE6 (the gene mutated in our arRP mouse model), and can prolong photoreceptor survival and visual response in other models of retinal degenerative diseases. We tested this hypothesis by treating a mouse model with autosomal dominant RP (adRP), originating from a genetic mutation in rhodopsin (*Rho^P23H^*), the most common cause of adRP (24, 25). We found that replenishment of the TCA cycle intermediates provided resilience against cell death to the photoreceptor neurons and prolonged visual function in this second preclinical model of RP.

## Results

### Metabolite profiling of the neural retina showed a decrease in TCA cycle intermediates in the arRP preclinical mouse model.

**Figure 1:**
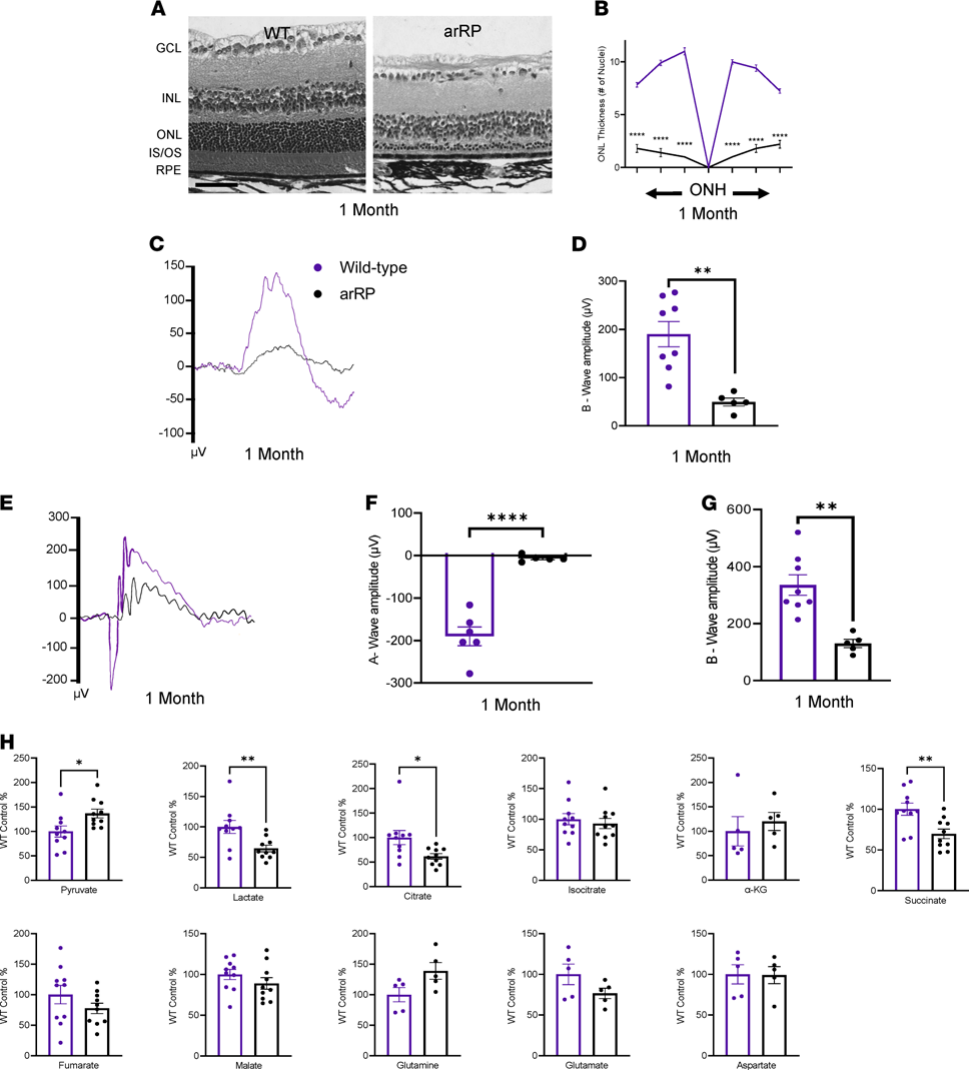
Metabolite profiling of the neural retina shows a decrease in TCA cycle intermediates in a model of arRP. (**A**) Retinal histology shows a loss of the photoreceptors (ONL) in the arRP mouse compared with a WT control. Scale bar: 50 μm. (**B**) Morphometric quantification of ONL thickness spanning from the ONH as measured by number of cell nuclei in each region of the retina for a WT mouse (purple) and an untreated arRP mouse (black). *n* = 3 eyes each group, with multiple counts per eye. Analyzed by multiple 2-tailed *t* tests with the Holm-Sidak method to correct for multiple comparisons. (**C**) Representative average traces from a scotopic ERG at a –1.1 log cd•s/m^2^ flash intensity. (**D**) Quantification of the b-wave amplitude shows a significant reduction in visual response for the arRP mice compared with WT controls. (**E**) Representative average traces from a scotopic ERG at a 2.5 log cd•s/m^2^ flash intensity. (**F**) Quantification of the a-wave amplitude shows a significant reduction in visual response for the arRP mice compared with controls. (**G**) Quantification of the b-wave amplitude shows a significant reduction in visual response for the arRP mice compared with controls. *n* = 8 eyes for WT, *n* = 5 eyes for arRP. (**H**) Mass spectrometry for the relative abundance of TCA cycle intermediates in the neural retinas from WT and arRP mice at 1 month of age. *n* ≥ 10 retinas. Data are represented as mean ± SEM. ERG and mass spectrometry data analyzed by student’s *t* test. *P < 0.05; **P < 0.01; ****P < 0.0001. TCA, tricarboxylic acid; arRP, autosomal recessive retinitis pigmentosa; ONL, outer nuclear layer; ONH, optic nerve head; GCL, ganglion cell layer; INL, inner nuclear layer; IS/OS, inner and outer segments; RPE, retinal pigment epithelium; ERG, electroretinogram.

The *Pde6*α*^D670G^* mouse model recapitulates the human arRP pathophysiology, with a loss of the majority of photoreceptor cell nuclei by 1 month of age (Figure 1, A and B). Electroretinography (ERG) displays a significant loss of both dim light (Figure 1, C and D) and bright light (Figure 1, E–G) scotopic visual function in the arRP preclinical mouse model by 1 month of age compared with WT controls. Because metabolic dysregulation has been shown to play a key role during age-related neurodegenerative diseases, we examined the arRP neural retina for the relative abundance of precise TCA cycle intermediates and additional metabolites at 1 month of age compared with WT controls (Figure 1H). Within the diseased RP environment at 1 month of age, neural retinas from the arRP mice showed a significant build-up of pyruvate and a significant reduction in lactate, citrate, and succinate compared with controls (Figure 1H). Therefore, during RP disease, the neural retina loses TCA cycle metabolic activity compared with healthy neural retinal tissue (Supplemental Figure 1; supplemental material available online with this article; https://doi.org/10.1172/jci.insight.150898DS1).

### Oral supplementation of α-KG prolongs photoreceptor cell survival and visual response while elevating citrate levels in the neural retina.

**Figure 2:**
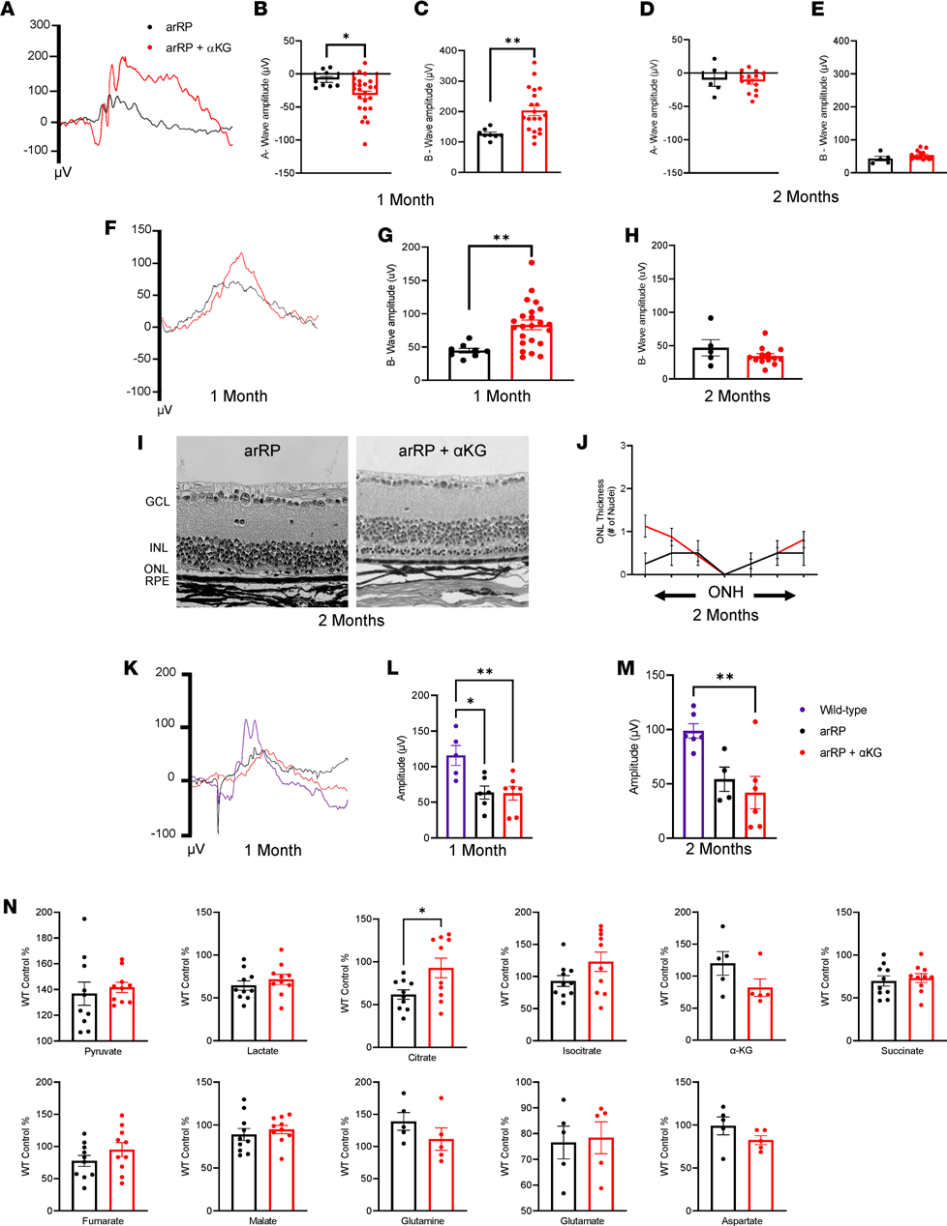
Supplementation with α-KG delays photoreceptor death, elevating citrate levels in the retina. (**A**) Representative ERG traces for untreated (black) and α-KG–treated (red) arRP mice at a 2.5 log cd•s/m^2^ flash intensity. (**B**) Quantification of a-wave and (**C**) b-wave amplitudes shows a significant increase in visual response in arRP mice treated with α-KG, (**D**) which was not significant at 2 months for either the a-wave (**E**) or b-wave. (**F**) Representative ERG traces at a –1.1 log cd•s/m^2^ flash intensity. (**G**) Quantification of the b-wave amplitude shows a significant increase in visual response in arRP mice treated with α-KG, (**H**) which was not significant at 2 months. *n* ≥ 5 eyes. (**I**) Histology of arRP retinas untreated or treated with α-KG. Scale bar: 50 μm. (**J**) Morphometric quantification of ONL thickness spanning from the ONH. *n* = 3 eyes each group, with multiple counts per eye. Analyzed by multiple 2-tailed *t* tests with the Holm-Sidak method to correct for multiple comparisons. (**K**) Photopic ERG traces at a 3.1 log cd•s/m^2^ flash intensity. (**L**) Quantification of the peak amplitude for WT mice (purple), untreated arRP mice, and arRP mice treated with α-KG at 1 (**M**) and 2 months of age. *n* ≥ 5 eyes. Analyzed by 1-way ANOVA followed by Tukey’s multiple comparisons test. (**N**) Mass spectrometry for the relative abundance of TCA cycle intermediates in the retinas from untreated arRP mice and α-KG–treated arRP mice at 1 month of age. *n* ≥ 10 retinas. Scotopic ERG and mass spectrometry analyzed by student’s *t* test. Data are represented as mean ± SEM. *P < 0.05; **P < 0.01. α-KG, α-ketoglutarate; ERG, electroretinogram; arRP, autosomal recessive retinitis pigmentosa; GCL, ganglion cell layer; INL, inner nuclear layer; ONL, outer nuclear layer; RPE, retinal pigment epithelium; ONH, optic nerve head; TCA, tricarboxylic acid.

We previously showed that α-KG supplementation can slow the disease progression of arRP in our preclinical mouse model at 1 month of age (21). We hypothesized that in the neural retina, α-KG supplementation was promoting TCA cycle activity. To test this hypothesis, we provided α-KG in the drinking water of the arRP mice beginning on P0. We compared late-stage disease progression (2 months of age) with the 1-month time point that has been previously published (21). We found that α-KG–treated arRP mice had a significant increase in bright light scotopic visual responses at 1 month of age compared with untreated arRP mice (Figure 2, A–C), but that the treatment’s efficacy in rescuing the visual response had diminished by 2 months of age (Figure 2, D and E). The significant increase in the a-wave scotopic ERG response at 1 month of age suggests that α-KG promoted the health of the photoreceptors (Figure 2B). Examination of dim light scotopic ERG responses showed a significant increase of the b-wave at 1 month of age, confirming a preservation of the rod photoreceptor function after α-KG supplementation (Figure 2, F and G). However, similar to the bright light scotopic ERG, there was no significant increase in visual response at the 2-month, late-stage disease time point (Figure 2H). Additionally, delivery of α-KG supplementation beginning at weaning age, P21, was unable to prolong visual function in the arRP mouse model, suggesting that the efficacy of α-KG is limited to early time points in disease progression (Supplemental Figure 2).

Histological analysis of α-KG–treated mice at 2 months of age compared with untreated arRP mice showed some retinal regions with prolonged neuronal cell survival in the outer nuclear layer (ONL) of the retina, representing the photoreceptor neurons (Figure 2, I and J). Immunostaining for rod and cone photoreceptors (rhodopsin and red/green opsin, respectively) confirmed an increase in rod photoreceptors after α-KG supplementation compared with untreated arRP mice at 1 month of age, and a retention of cone photoreceptors at both 1 and 2 months of age. However, there was abnormal morphology present for the cone photoreceptors in both the untreated and treated arRP mice (Supplemental Figure 3, A and B).

Photopic ERG was used to analyze the cone cell function at both 1 and 2 months of age (Figure 2, K–M). The cone cell response was significantly diminished in arRP mice compared with WT controls, with no difference between untreated mice and those treated with α-KG. The cone cell response was similar at both 1 and 2 months of age for all groups, suggesting that there was a reduction in cone cell function by 1 month of age that was not altered by α-KG supplementation, but that the remaining cone cell responses at 1 month of age persisted through at least 2 months of age (Figure 2, L–M). Overall, α-KG supplementation provided resilience to the photoreceptor neurons against cell death to delay, but not cure, arRP disease progression.

To investigate the mechanism for the therapeutic effect provided by α-KG supplementation at 1 month of age, we collected P28 neural retina samples from untreated and α-KG–treated arRP mice. We used mass spectrometry to analyze the precise changes in TCA cycle intermediates and metabolites that support TCA cycle activity, and found that supplementation of α-KG led to a significant increase in citrate levels, to approximately WT abundance, but no significant difference in succinate, fumarate, or other metabolites (Figure 2N). These data suggest that the systemic delivery of α-KG is causing shifts in the TCA cycle in the reverse direction, favoring the conversion of α-KG into citric acid in the neural retina. This mechanism of promoting reductive carboxylation of the TCA cycle is important to provide citrate for cell growth and viability, as has been shown in studies examining tumor cell growth and hypoxic conditions. Interestingly, in the eye, reductive carboxylation of the TCA cycle has been shown to be a main metabolic pathway for the retinal pigment epithelial (RPE) cells, but is not known to be a main pathway for the neural retina (19). We therefore hypothesized that enhancing citric acid production in a diseased neural retinal environment may provide this resilience to the photoreceptors against cell death.

### Oral supplementation of citrate prolongs photoreceptor cell survival and visual function in the preclinical model of arRP.

**Figure 3:**
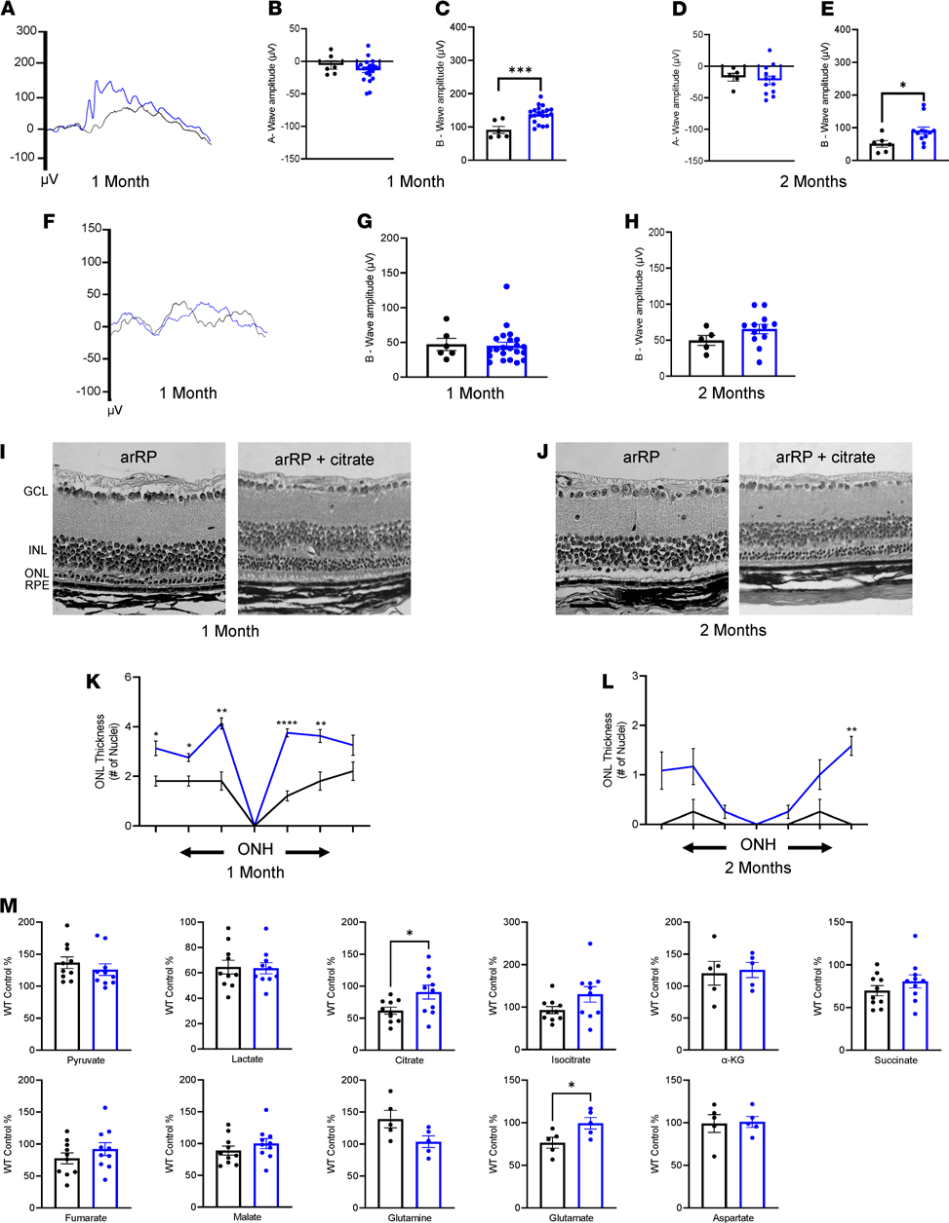
Citrate supplementation prolongs photoreceptor cell survival and visual function in the preclinical model of arRP. (**A**) Representative average ERG traces for arRP mice (black) and arRP mice treated with citrate (blue) recorded at a 2.5 log cd•s/m^2^ flash intensity. (**B**) Quantification of the a-wave (**C**) and b-wave amplitude at 1 month of age shows citrate treatment causing a rescue in the b-wave response but no significant increase in a-wave response. (**D**) A similar trend was seen at 2 months of age, with no significant increase in a-wave amplitude (**E**) but a significant rescue of the b-wave response of arRP mice treated with citrate. (**F**) Representative average ERG traces for arRP mice and arRP mice treated with citrate recorded at a –1.1 log cd•s/m^2^ flash intensity. (**G**) Quantification of the b-wave amplitudes at 1 month (**H**) and 2 months of age. *n* ≥ 5 eyes. (**I**) Histology of the arRP retinas treated with citrate compared with untreated arRP retinas at both 1 and (**J**) 2 months of age. Scale bar: 50 μm. (**K** and **L**) Morphometric quantification of ONL thickness spanning from the ONH. *n* = 3 eyes each group, with multiple counts per eye. Analyzed by multiple 2-tailed *t* tests with the Holm-Sidak method to correct for multiple comparisons. (**M**) Mass spectrometry for the relative abundance of TCA cycle intermediates in retinas from untreated arRP mice and citrate-treated arRP mice at 1 month of age. *n* ≥ 10 retinas. ERG and mass spectrometry data analyzed by student’s *t* test. Data are represented as mean ± SEM. *P < 0.05; **P < 0.01; ***P < 0.001; ****P < 0.0001. ERG, electroretinogram; arRP, autosomal recessive retinitis pigmentosa; GCL, ganglion cell layer; INL, inner nuclear layer; ONL, outer nuclear layer; RPE, retinal pigment epithelium; ONH, optic nerve head; TCA, tricarboxylic acid.

To test this hypothesis, we delivered citrate to the arRP mice via drinking water beginning on P0 and analyzed these mice for the potential of citrate supplementation to provide a similar, or enhanced, resilience to the photoreceptor neurons against cell death compared with that of α-KG supplementation. Evaluation of visual function at 1 month of age by bright light scotopic ERG showed that the citrate-treated mice had a significant response to visual stimuli — with an average b-wave of 138 μV — compared with the untreated arRP mice with a b-wave of 91 μV (Figure 3, A and C). However, a-wave and dim light scotopic ERG responses showed no change in rod function between untreated and citrate-treated arRP mice at 1 month of age (Figure 3, B, F, and G) or at 2 months of age (Figure 3, D and H).

Histology at 1 month of age showed that citrate treatment led to significantly improved neuronal cell survival of the photoreceptors, and immunostaining displayed both rod and cone cells with a healthier morphology compared with untreated arRP mice (Figure 3, I and K, and Supplemental Figure 3, A and C). At 2 months of age, the arRP mice treated with citrate had continued significant rescue of visual function shown on bright light scotopic ERG (Figure 3E), as well as some significant rescue in the number of photoreceptors present within the neural retina (Figure 3, J and L). Thus, supplementation with citrate alone is able to provide resilience to the photoreceptor neurons against cell death and prolong visual function, for a longer duration than that of α-KG supplementation.

Based on these results, we hypothesized that citrate supplementation was acting via reductive carboxylation of the TCA cycle to promote production of biomass building blocks, such as fatty acids, and therefore would not have a significant effect on other TCA cycle metabolites. We performed mass spectrometry to test for the relative abundance of TCA cycle metabolites and additional metabolites that support the TCA cycle in our arRP untreated neural retinas compared with neural retinas from mice treated with citrate at 1 month of age. The metabolite profiling of citrate-treated mice showed a significant increase in citrate, to approximately WT abundance (Figure 3M), similar to the results found after α-KG supplementation (Figure 2N). We did additionally found that citrate supplementation significantly elevated glutamate levels, to approximately WT abundance, but that there were no significant changes in other TCA cycle-related metabolites (Figure 3M). Thus, the protective effects of both the citrate and the α-KG treatment in rescuing visual function and prolonging neuronal cell survival were likely promoting synthesis of biomass building blocks to preserve photoreceptor cells in their diseased retinal environment.

### Oral supplementation of additional metabolites that feed into the TCA cycle provide resilience against cell death for the photoreceptors and prolong visual function in the arRP preclinical mouse model.

**Figure 4:**
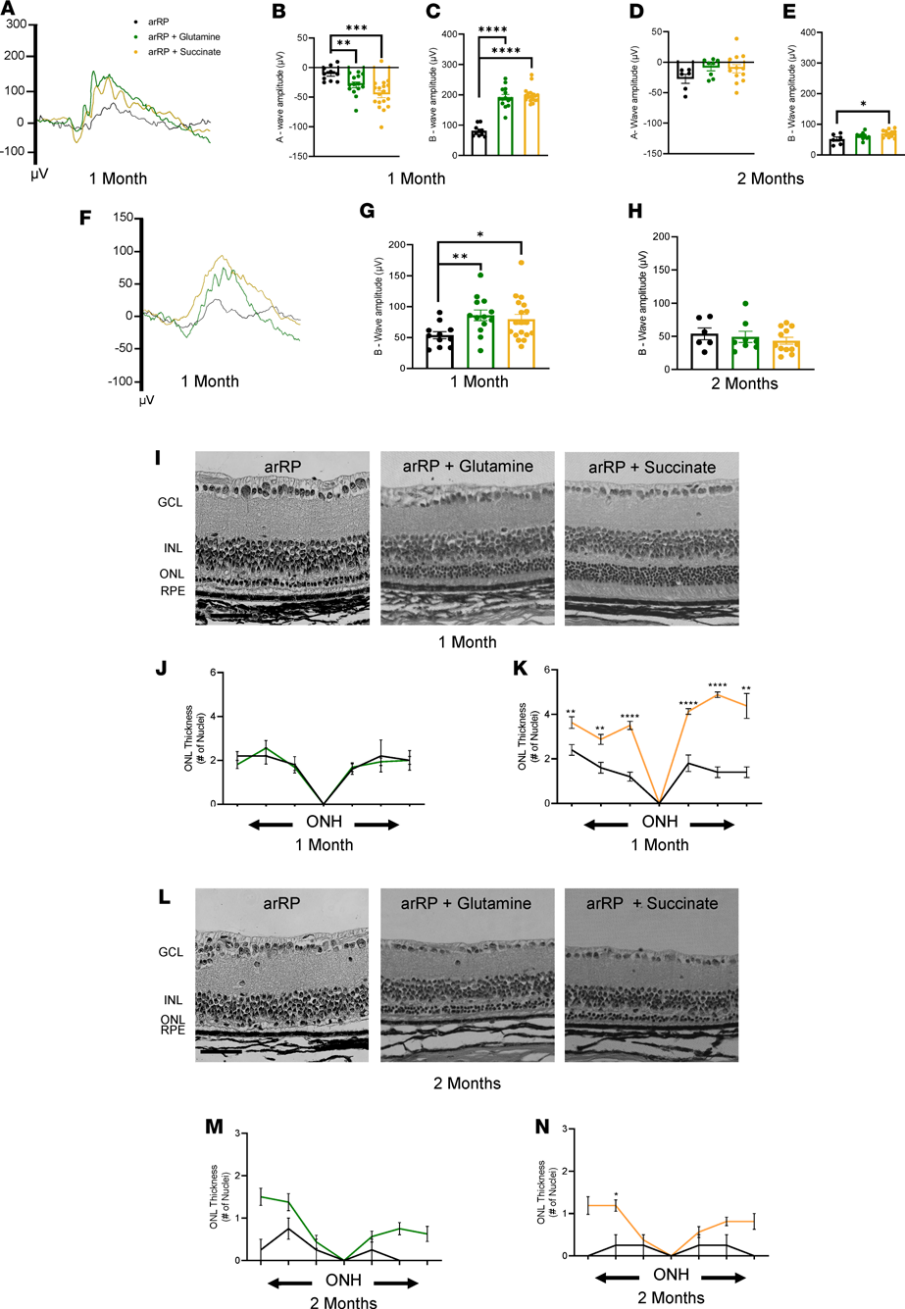
Succinate supplementation provides resilience against cell death for the photoreceptors and prolongs visual function. (**A**) Representative average ERG traces for untreated arRP mice (black) and arRP mice treated with glutamine (green) or succinate (yellow) recorded at a 2.5 log cd•s/m^2^ flash intensity. (**B**) Quantification of the a-wave (**C**) and b-wave amplitude for both glutamine-treated arRP mice and succinate-treated arRP mice compared with untreated arRP mice. (**D**) By 2 months of age, no significant change was detected in the a-wave response for either succinate or glutamine treatment, (**E**) but succinate-treated arRP mice showed an increase in b-wave response. (**F**) Representative average ERG traces for untreated arRP mice and arRP mice treated with glutamine or succinate recorded at a –1.1 log cd•s/m^2^ flash intensity. (**G**) Quantification of the b-wave amplitude shows a significant increase for arRP mice treated with glutamine or succinate at 1 month (**H**) but shows no detectable increase by 2 months of age. *n* ≥ 5 eyes. (**I**) Histology of the arRP retinas treated with either glutamine or succinate compared with untreated arRP retinas at 1 (**L**) and 2 months of age. Scale bar: 50 μm. (**J** and **K**) Morphometric quantification of ONL thickness spanning from the ONH at 1 (**M** and **N**) and 2 months of age. *n* = 3 eyes each group, with multiple counts per eye. ONL Thickness was analyzed by multiple 2-tailed *t* tests with the Holm-Sidak method to correct for multiple comparisons. ERG data were analyzed by student’s *t* test. Data are represented as mean ± SEM. *P < 0.05; **P < 0.01; ***P < 0.001; ****P < 0.0001. ERG, electroretinogram; arRP, autosomal recessive retinitis pigmentosa; GCL, ganglion cell layer; INL, inner nuclear layer; ONL, outer nuclear layer; RPE, retinal pigment epithelium; ONH, optic nerve head.

Reductive glutamine metabolism promotes conversion of α-KG to citrate for fatty acid synthesis, and has been shown to be activated in cells when the α-KG to citrate ratio is elevated (26). Therefore, we hypothesized that if the resilience to the photoreceptors against cell death during RP disease progression is due to α-KG’s conversion to citrate, then treatment with glutamine will likely have a similar effect to that of α-KG supplementation. Additionally, although we did not see an elevation of succinate after supplementation with either α-KG or citrate, succinate is a direct metabolite of the TCA cycle and can be utilized by the photoreceptors to produce citrate and promote TCA cycle activity during RP disease progression. We treated our arRP preclinical mice with either glutamine or succinate in the drinking water beginning on P0, and tested them for photoreceptor cell survival and visual function at both 1 and 2 months of age. Representative bright light scotopic ERG traces of both treatment groups showed a significantly increased visual response compared with the untreated arRP mice at 1 month of age (Figure 4A). Quantification of the ERG b-waves showed an average b-wave for glutamine and succinate as 192 and 199 μV, respectively, significantly higher than the 81 μV average of the untreated arRP mice at 1 month of age (Figure 4C). Quantification of the scotopic ERG b-wave at 2 months of age showed that the succinate treatment provided significantly higher visual function compared with untreated arRP mice (Figure 4E), but was slightly lower in amplitude than that from citrate supplementation (Figure 3E). Because the a-wave was also significant after treatment with both glutamine and succinate at 1 month of age (Figure 4B), dim light scotopic ERG was used to evaluate rod photoreceptor function. Both treatment groups had higher b-wave responses compared with untreated controls (Figure 4, F and G); however, no significant difference in this dim light response or a-wave response was detected at 2 months of age, indicating a loss of rod photoreceptor function over time (Figure 4, D and H).

Histological analysis of the retina at 1 month of age showed that treatment with succinate significantly improved neuronal cell survival in all regions of the retina, whereas glutamine treatment showed a high variation and did not significantly rescue the photoreceptor cells (Figure 4, I–K). Some of the analyzed eyes treated with succinate showed not only significant rescue of photoreceptors in the ONL, but also a thicker, more intact layer of the inner and outer segments at 1 month of age (Figure 4I and Supplemental Figure 3D). Histological analysis at 2 months of age showed regions of succinate treatment, but not glutamine treatment, having improved neuronal cell survival at this late stage of disease (Figure 4, L–N). Overall, glutamine supplementation provided a similar, slightly lessened, effect to that of α-KG supplementation, whereas directly targeting the TCA cycle with succinate provided a longer lasting resilience to the photoreceptor neurons, similar to that of citrate supplementation.

### Metabolite supplementation improved neuronal cell survival during a state of stress on the retina.

**Figure 5:**
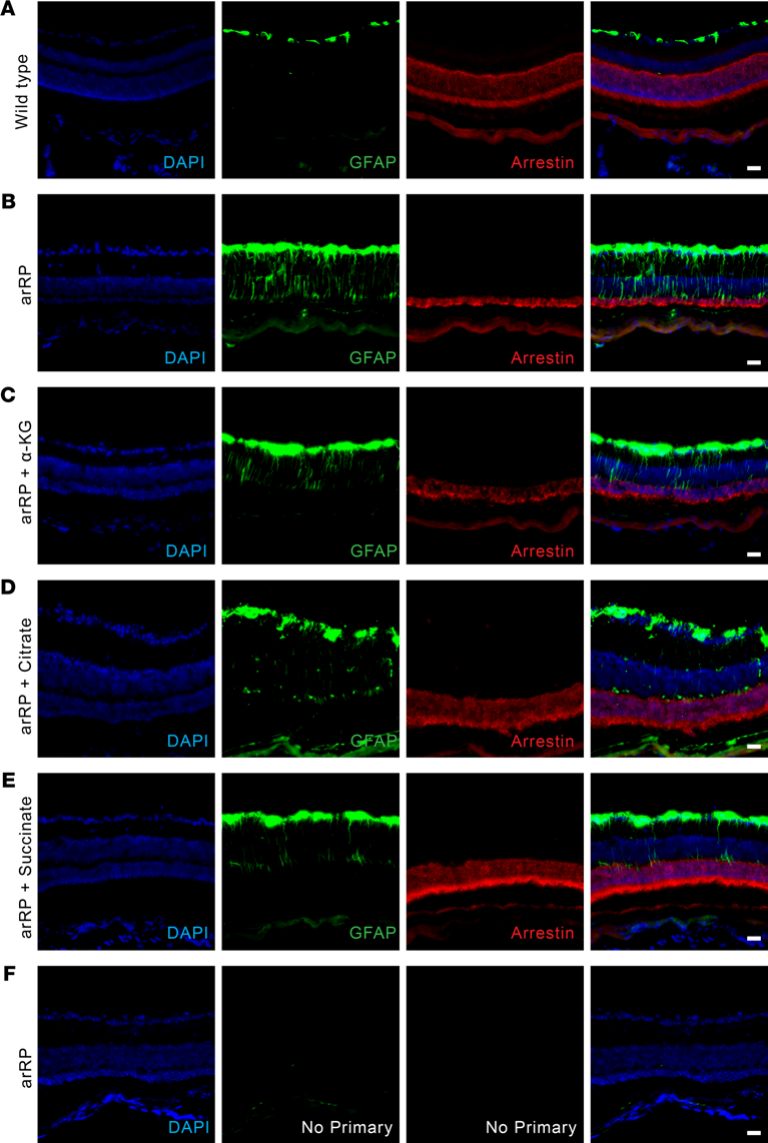
Metabolite supplementation improves neuronal cell survival during a state of stress on the retina. GFAP staining shows Muller glial cell activation in the mouse neural retina of a (**A**) WT C57BL/6J mouse, (**B**) untreated arRP mouse, (**C**) arRP mouse treated with α-KG, (**D**) arRP mouse treated with citrate, and (**E**) arRP mouse treated with succinate, all at 1 month of age. (**F**) An untreated arRP mouse retina with no primary stain shows no nonspecific binding or autofluorescence with the secondary antibodies. Blue, DAPI (nuclei); green, GFAP; red, arrestin (photoreceptors). Scale bar: 100 μm. arRP, autosomal recessive retinitis pigmentosa; GFAP, glial fibrillary acidic protein; α-KG, α-ketoglutarate.

Glial activation happens when the retina is in a state of stress, such as occurs during photoreceptor degeneration and RP disease progression, where Muller glia express glial fibrillary acidic protein (GFAP) immunoreactivity (27). When retinal sections of a healthy eye are stained for GFAP signal, only a small layer of the Muller glia cell nuclei are shown to express GFAP around the ganglion cell layer (Figure 5A), with no GFAP expression detected without the primary antibody (Figure 5F). In the arRP mouse retina at 1 month of age, the entire processes of the Muller glial cells express GFAP in response to the retinal stress from the disease state (Figure 5B). We examined the neural retinas of arRP mice that had been treated with α-KG, citrate, or succinate, all 3 being metabolites directly involved in the TCA cycle activity. We found that at 1 month of age, there was still glial activation and therefore stress present within the retina, despite the slower progression of the disease in the treatment groups (Figure 5, C–E). These data show that targeting the TCA cycle by systemic delivery of metabolites was not corrective for the diseased environment, but provided resilience to the photoreceptor cells against cell death and prolonged their functionality during RP progression, within the stressed and unhealthy neural retinal environment.

### Replenishment of TCA cycle metabolites, α-KG and citrate, provide resilience to the photoreceptor neurons against cell death and prolongs visual function during RP disease unrelated to the causative genetic mutation.

**Figure 6:**
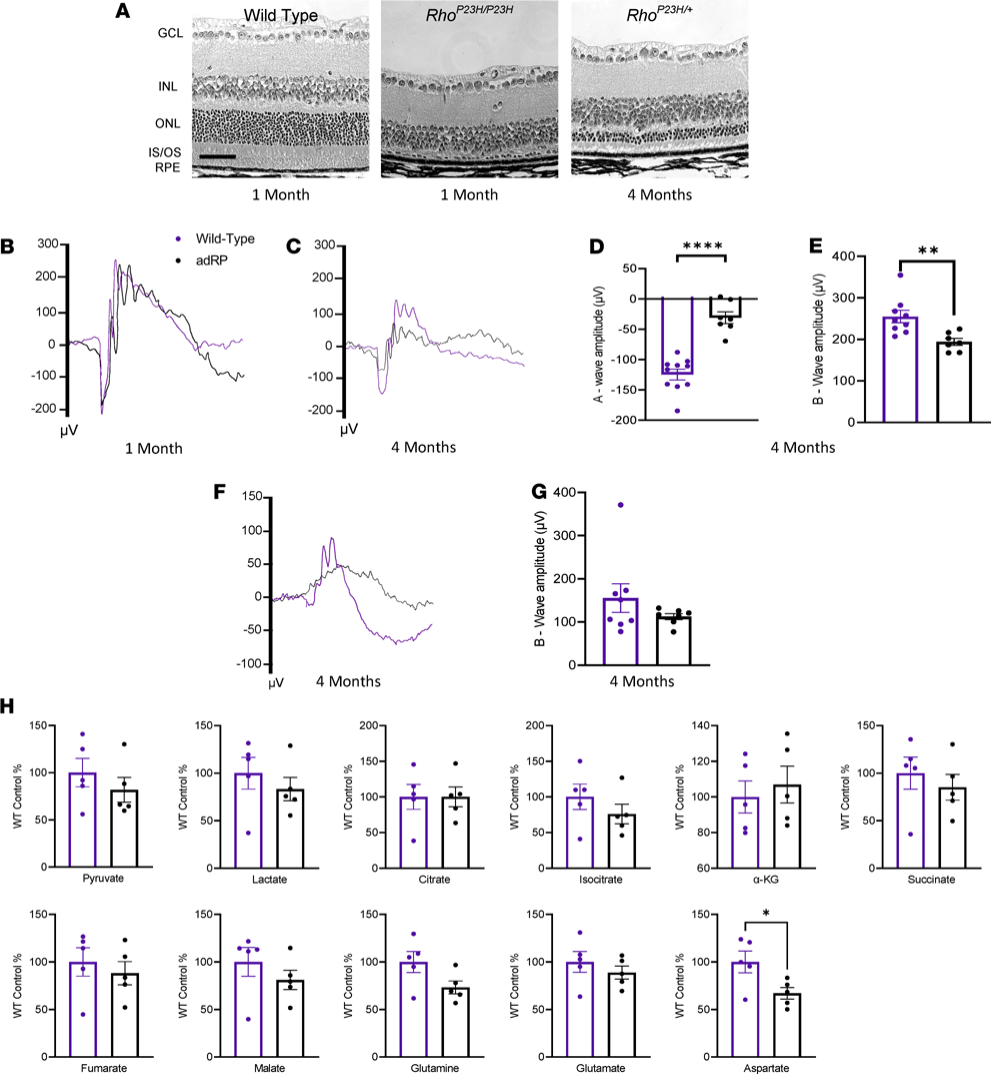
Disease pathophysiology and metabolic analysis of an autosomal dominant preclinical model of RP. (**A**) Histology of the retina for a WT control retina, a homozygous adRP mouse at 1 month of age, and the heterozygous adRP mouse at 4 months of age. Scale bar: 50 μm. (**B**) Representative average ERG traces from a 2.5 log cd•s/m^2^ flash intensity setting shows no difference in visual function in the heterozygous adRP mouse model (black) at 1 month of age compared with a WT control (purple), (**C**) but shows a reduction in visual response in the adRP mouse by 4 months of age. (**D**) Quantification at 4 months of age shows a significant reduction in visual response for the adRP mice compared with WT controls in both the a-wave (**E**) and b-wave amplitudes. (**F**) Representative average ERG traces recorded at a –1.1 log cd•s/m^2^ flash intensity. (**G**) Quantification of the b-wave response shows a reduction in the adRP mice compared with the WT mice. *n* ≥ 8 eyes. (**H**) Mass spectrometry for the relative abundance of TCA cycle intermediates in the retinas from WT and adRP mice at 3 months of age. *n* = 10 retinas. ERG and mass spectrometry data analyzed by student’s *t* test. Data are represented as mean ± SEM. *P < 0.05; **P < 0.01; ****P < 0.0001. adRP, autosomal dominant retinitis pigmentosa; GCL, ganglion cell layer; INL, inner nuclear layer; ONL, outer nuclear layer; IS/ OS, inner and outer segments; RPE, retinal pigment epithelium; ERG, electroretinogram; TCA, tricarboxylic acid.

Because replenishment of the TCA cycle intermediates prolonged photoreceptor cell survival and visual function in a diseased neural retinal environment, we hypothesized that treatment with these metabolites would provide therapeutic benefit unrelated to the causal genetic mutation for the underlying disease state. For RP disease, adRP makes up approximately 30% of the cases, and the main causal mutation for approximately 10% of adRP cases is the p.P23H mutation in rhodopsin (RHO) (25, 28). To test our hypothesis, we utilized the *Rho^P23H^* preclinical mouse model of adRP (24, 25). Compared with WT controls, *Rho^P23H/P23H^* mice had a rapid degeneration, with nearly all photoreceptor neurons lost by 1 month of age (Figure 6A). In the heterozygous state, the mice had a slower disease progression, with a loss of the ONL similar to that of the *Pde6*α*^D670G^* arRP mouse model by 4 months of age (Figure 6A). These *Rho^P23H/+^* mice show similar bright light scotopic ERG visual responses to WT control mice at 1 month of age (Figure 6B), with a significant reduction in visual response by 4 months of age (Figure 6, C–G).

**Figure 7:**
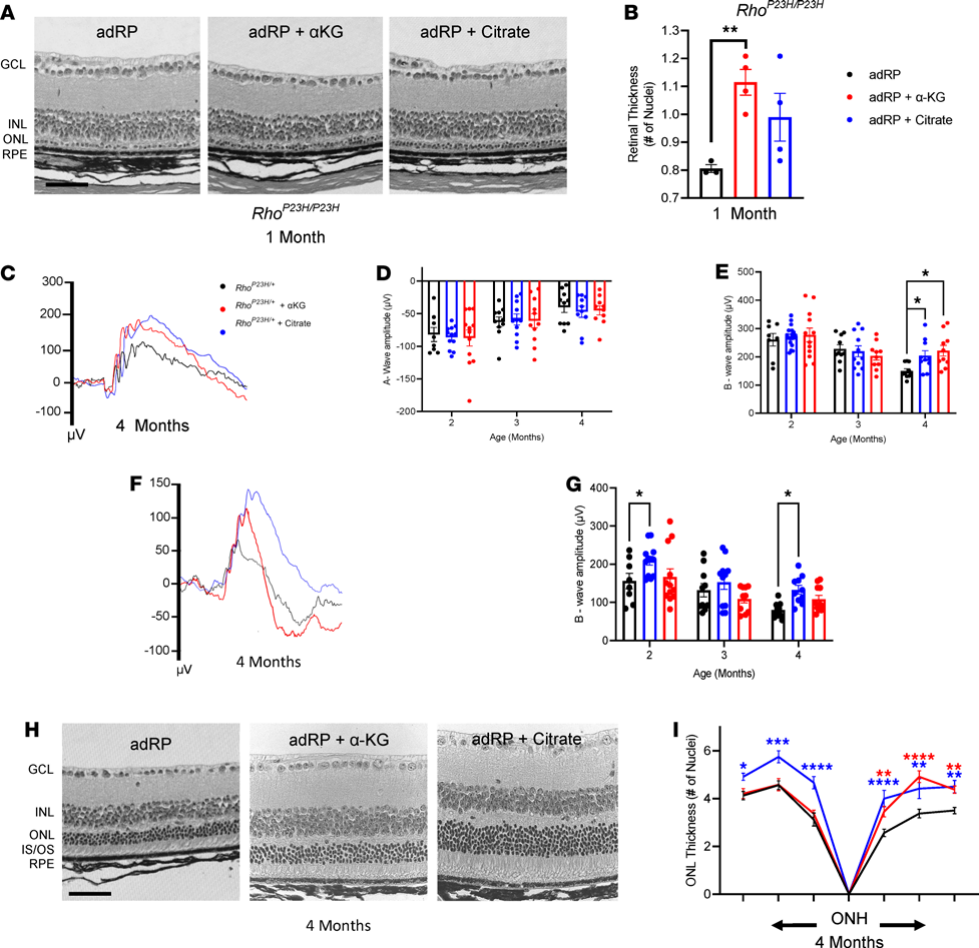
Citrate and α-KG provide photoreceptor resilience against death and prolong visual function during adRP disease. (**A**) Histology of the untreated and the adRP retina treated with either α-KG or citrate. (**B**) Quantification of photoreceptor nuclei thickness, spanning from the ONH to either direction. *n* ≥ 3 eyes. (**C**) Representative ERG traces for adRP mice (black) and adRP mice treated with α-KG (red) or citrate (blue) at a 2.5 log cd•s/m^2^ flash intensity. (**D**) Quantification of the a-wave and (**E**) b-wave amplitude. (**F**) Representative ERG traces at a –1.1 log cd•s/m^2^ flash intensity. (**G**) Quantification of the b-wave amplitude. *n* ≥ 8 eyes. (**H**) Histology of the untreated and the adRP retina treated with α-KG or citrate. Scale bar: 50 μm. (**I**) Morphometric quantification of ONL thickness spanning from the ONH. *n* ≥ 3 eyes. Statistical analysis by 1-way ANOVA (**B**) or 2-way ANOVA with Tukey’s multiple comparisons test (**D**, **E**, **G**, and **I**). Data are represented as mean ± SEM. *P < 0.05; **P < 0.01; ***P < 0.001; ****P < 0.0001. Red and blue asterisks reflect significance of α-KG–treated and citrate-treated mice, respectively, in comparison with untreated controls. α-KG, α-ketoglutarate; adRP, autosomal dominant retinitis pigmentosa; GCL, ganglion cell layer; INL, inner nuclear layer; ONL, outer nuclear layer; IS/OS, inner and outer segments; RPE, retinal pigment epithelium; ONH, optic nerve head; ERG, electroretinogram.

We examined the adRP neural retina for the relative abundance of TCA cycle intermediates, and metabolites that support the TCA cycle, at 3 months of age (early in photoreceptor cell degeneration/disease progression) compared with WT controls (Figure 6H). Within the diseased RP environment at 3 months of age, neural retinas from the adRP mice showed a significant reduction in aspartate, which can feed into oxaloacetate in the TCA cycle, but no other significant changes were found in the TCA cycle metabolites compared with controls (Figure 6H). Although the overall TCA cycle activity was not significantly reduced in the adRP preclinical mouse model, we hypothesized that enhancing TCA cycle activity may still promote photoreceptor resilience against cell death during disease progression. Therefore, we used these adRP mice to evaluate the broad applicability of α-KG and citrate supplementation to promote photoreceptor resilience against neurodegeneration, following our experimental protocol for the arRP mouse model. Histological analysis of the retina of homozygous *Rho^P23H/P23H^* mice at 1 month of age displayed a significant rescue of photoreceptor cell nuclei in the mice treated with α-KG compared with untreated controls (Figure 7, A and B). Citrate supplementation displayed a similar thickness of the ONL to that of the α-KG–treated mice; however, in this rapid degeneration model, the number of nuclei was not significant compared with WT controls (Figure 7, A and B).

To test for visual function, heterozygous *Rho^P23H/+^* mice were treated with either α-KG or citrate and followed over time by ERG. Bright light scotopic ERG showed a significant increase in visual function as measured by the b-wave amplitude in both α-KG–treated and citrate-treated adRP mice at 4 months of age compared with WT controls (Figure 7, C and E). Additionally, there was no statistically significant difference in visual response (i.e., no decline) in adRP mice treated with α-KG from 2 to 4 months of age, and no statistically significant change in visual response for adRP mice treated with citrate from 3 to 4 months of age. Although the a-wave was not significantly different between untreated and treated mice (Figure 7D), dim light scotopic ERG showed a significant increase in the b-wave amplitude of adRP mice treated with citrate at both 2 and 4 months of age (Figure 7, F and G), indicating increased rod photoreceptor function after citrate supplementation.

Histological analysis showed a significant increase in the ONL thickness in both the α-KG and citrate-treated adRP mice at 4 months of age, with the α-KG treatment being limited to only portions of the retina, but the citrate treatment showing as significant in all regions of the retina (Figure 7, H and I). A thicker and more robust layer of the inner and outer segments of the photoreceptors can be seen in the citrate-treated adRP mice (Figure 7H and Supplemental Figure 4). Therefore, the replenishment of either α-KG or citrate was able to prolong photoreceptor cell survival and visual function in the arRP and adRP preclinical models, carrying mutations in 2 different genes leading to RP disease pathology.

**Figure 8:**
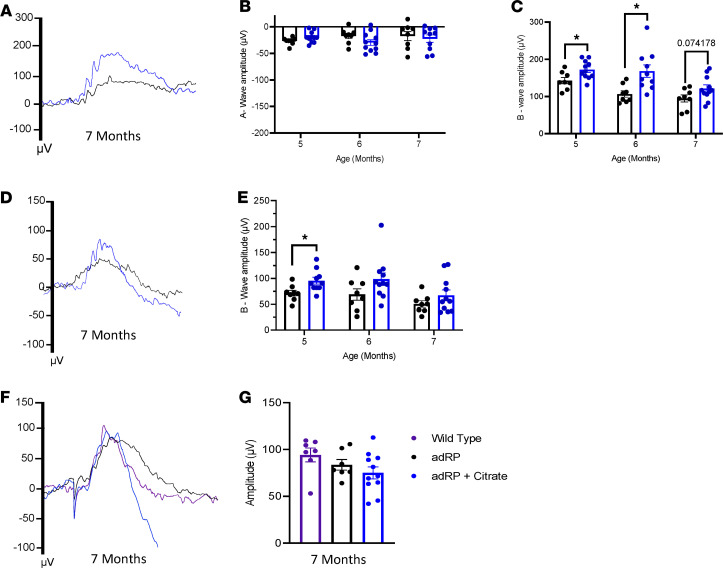
Citrate provides photoreceptor resilience against death and prolongs function long-term in adRP. (**A**) Representative ERG traces for adRP mice (black) and adRP mice treated with citrate (blue) at a 2.5 log cd•s/m^2^ flash intensity. (**B**) Quantification of the a-wave and (**C**) b-wave amplitudes over time. (**D**) Representative ERG traces at a –1.1 log cd•s/m^2^ flash intensity. (**E**) Quantification of the b-wave amplitudes over time. (**F**) Photopic ERG traces for WT mice (purple) and untreated and citrate-treated adRP mice at a 3.1 log cd•s/m^2^ flash intensity. (**G**) Quantification of the peak amplitudes. *n* ≥ 7 eyes. Statistical analysis by 1-way ANOVA (**G**) or multiple 2-tailed *t* tests with the Holm-Sidak correction (**B**, **C**, and **E**). Data are represented as mean ± SEM. *P < 0.05. adRP, autosomal dominant retinitis pigmentosa; ERG, electroretinogram

Because the citrate treatment prolonged visual function in the arRP model to 2 months of age (Figure 3E) and showed efficacy through 4 months of age in the adRP model (Figure 7, C–I), we hypothesized that it would prolong visual function in the adRP model beyond 4 months of age. Scotopic ERG responses showed an increase in bright light b-wave amplitudes for citrate-treated adRP mice at both 5 and 6 months of age (Figure 8, A–C), and an increase in dim light b-wave amplitudes at 5 months of age (Figure 8, D and E). By 7 months of age, the retinas were greatly degenerated and showed a higher variation in visual response after citrate supplementation, although some mice displayed elevated visual responses compared with untreated adRP mice on both dim and bright light scotopic ERG at 7 months of age (Figure 8, A–E). Photopic ERGs showed no change in cone function between WT controls, untreated adRP mice, and citrate-treated adRP mice at 7 months of age, reflecting a retention of the cone photoreceptors through this time (Figure 8, F and G). Overall, the systemic delivery of citrate was found to provide prolonged photoreceptor cell function in both the arRP and adRP mouse models.

## Discussion

Age-related neurodegenerative diseases, such as RP, severely impair patients’ quality of life, as they are no longer able to read, drive, or perform independent daily activities. For the majority of these diseases, treatment to delay photoreceptor cell death and preserve visual response is unavailable. RP consists of a group of inherited retinal disorders with high genetic heterogeneity, which can be caused by individual mutations in more than 60 genes. Therefore, it is likely that there are common mechanisms underlying photoreceptor degeneration in RP regardless of the individual genetic mutation. Recent research studies provide accumulating evidence linking neurodegeneration to energy metabolism, particularly in age-related neurological disorders like Huntington’s, Alzheimer’s, and Parkinson’s disease (29). Because the retina is one of the most metabolically demanding tissues in the body, targeting retinal metabolism is a potential therapeutic approach for retinal degenerative diseases regardless of their underlying genetic cause (6–8).

In our current study, we examined a preclinical mouse model of arRP at mid-stage of disease (1 month of age) and late stage of disease (2 months of age). We found that TCA cycle activity was reduced in neural retinas undergoing degeneration from RP disease compared with that of healthy, WT neural retinal tissue. We have previously determined that oral supplementation of α-KG can prolong photoreceptor cell survival and visual function for 1 month in the arRP model (21). Here, we identified the effect of α-KG supplementation on precise TCA cycle metabolites, showing that α-KG supplementation significantly elevated citrate within the neural retinal tissue. It is likely that α-KG supplementation promoted citrate production to increase the synthesis of biological precursors, such as lipids, to promote the neuronal cell health of the photoreceptors. As recent studies have defined the “metabolic ecosystem” that exists within the retina, it is also possible that the α-KG supplementation may impact other cells (RPE, Muller glia, etc.) within the neural retina to support the photoreceptors during disease. Additionally, we tested α-KG supplementation at a mid-stage of disease (P21) and found no significant rescue of visual function. At this time, the retina had undergone substantial degeneration before the onset of treatment, and it may have been too late to shift metabolism within the neural retina to support the degenerating rod photoreceptor neurons. However, it is also possible that our early treatment on P0 impacted postnatal retinal development and altered overall metabolism in the neural retina to delay RP disease progression.

In support of our hypothesis that promoting TCA cycle activity to allow for the production of citrate is beneficial to photoreceptor cell health during RP disease progression, the treatment of our preclinical arRP mouse model with citrate alone was able to provide resilience against photoreceptor cell death and prolonged visual function, which had a longer duration than the effect of α-KG supplementation. Elevation of citrate within the neural retina can promote the synthesis of biosynthetic precursors, such as lipids, that are needed for photoreceptor cell health during disease. Proper lipid metabolism is particularly important in the case of neuronal cell health because lipids are known to play a role in neurodegeneration and loss of synaptic plasticity (30). Photoreceptors are constantly tasked with generating lipids to replenish the membranes of rod outer segments, making anabolism of lipids a key process in the photoreceptors (31). We did find that citrate supplementation significantly increased the neurotransmitter glutamate within the neural retina at 1 month of age, which could promote enhanced signaling to the inner retinal cells from the photoreceptors that have not yet degenerated.

We also found that glutamine supplementation followed the α-KG therapeutic effect, as would be expected if glutamine is undergoing glutaminolysis to α-KG. Interestingly, succinate supplementation provided a highly significant survival of the photoreceptors during RP disease progression. Studies have shown that the neural retina produces succinate that is then shuttled into the RPE and oxidized. This occurs through the reversal of succinate dehydrogenase (SDH), favoring the formation of succinate from fumarate (20). In our study, we found no significant difference in succinate or fumarate levels after either α-KG or citrate supplementation, approaches which provided resilience against cell death to the photoreceptors during RP disease progression. However, as metabolite delivery was provided orally, it is possible that succinate is acting within the RPE or another cell type to promote resilience of the photoreceptors against cell death. With succinate’s unique role in the retina and SDH being the only enzyme to function in both the TCA cycle and the electron transport chain, succinate may be functioning in a separate mechanism from α-KG and citrate, to preserve the photoreceptor cells.

Because the metabolite supplementation provided therapeutic efficacy within a diseased retinal environment, as indicated by glial activation in the neural retina, the enhancement of TCA cycle activity is likely to be beneficial to the photoreceptor neurons regardless of the specific causative genetic mutation leading to the disease pathophysiology. Supporting this hypothesis, we found that replenishment of these TCA cycle intermediates provided resilience to the photoreceptor neurons against cell death and prolonged visual function in an adRP preclinical mouse model, supporting the broad applicability of this approach to promote TCA cycle activity in the neural retina during disease progression. This treatment efficacy was noted both in the rapidly degenerating homozygous adRP mice (with almost a full loss of photoreceptors by 1 month of age) the slow-progressing heterozygous adRP mice, with mid-stage of disease occurring at approximately 4 months of age.

In this study, the variability in visual responses after metabolite supplementation may be caused by the amount of drinking water consumed per mouse. However, the oral supplementation doses were chosen based on previously published literature (21, 32–34), to provide the highest doses without toxicity to the animals. To consider this supplementation as a clinical therapeutic approach, both the dose and timing of delivery will need to be investigated. This study focused on metabolic supplementation as a means to understand the mechanisms for promoting photoreceptor cell survival within a diseased retinal state over time, in vivo, rather than as a direct therapeutic option. We have identified that replenishing TCA cycle intermediates affected photoreceptor cell resilience against cell death during disease in 2 in vivo model systems. This approach suggests that therapeutic targets to promote TCA cycle activity in the photoreceptor neurons delayed disease progression while allowing the cell to adapt to the appropriate metabolic needs for its environment.

## Methods

### Mouse lines and husbandry.

C57BL/6J-*Pde6*α*^nmf363/nmf363^*, with a D670G mutation, herein referred to as arRP mice, were obtained from Patsy Nishina at the Jackson Laboratory (Bar Harbor, Maine, USA). C57BL/6J-*Rho^P23H/P23H^*, herein referred to as adRP mice, were obtained from the Jackson Laboratory. The arRP and the adRP mice are coisogenic in the C57BL/6J (B6) background; therefore, age-matched B6 mice were used as experimental controls. Mice were bred and maintained at the facilities of the UT Southwestern Medical Center. Animals were kept on a light–dark cycle (12 hour–12 hour). Food and water were available ad libitum throughout the experiment, whether treated or untreated. Treatments were provided to the mice via the drinking water beginning on P0 or P21, and treatment groups consisted of litters of mice, with both female and male mice present in every group. α-KG (α-ketoglutaric acid, MilliporeSigma) was provided at a concentration of 10 mg/mL in the mouse drinking water. Glutamine (L-glutamine, MilliporeSigma) was provided at a concentration of 10 mg/mL in the mouse drinking water. Citrate was provided by combining Potassium Citrate Tribasic Monohydrate (MilliporeSigma) at a concentration of 18 mg/mL and Citric Acid (MilliporeSigma) at a concentration of 12.8 mg/mL in the mouse drinking water. Succinate (Succinic Acid, MilliporeSigma) was provided at a concentration of 0.8 mg/mL in the mouse drinking water (21, 32–34). All water treatments were provided in red light protective mouse water bottles and changed weekly during the duration of the experiment following approved institutional guidelines.

### Mouse retina dissection.

Retinas were collected as described previously (35). Briefly, scleral tissue posterior to the limbus was grasped with 0.22 forceps and a microsurgical blade was used to make a linear incision in the cornea from limbus to limbus. A fine curved needle holder was inserted behind the lens toward the posterior aspect of the globe. The needle holder was partially closed and pulled forward, pushing the lens through the corneal incision while leaving the eye wall intact. The fine curved needle holder was placed as far posterior to the globe as possible, near the optic nerve. The needle holder was partially closed and pulled forward, pushing the retina forward through the corneal incision. The retina was quickly rinsed in 1x PBS and then placed in a screw cap tube and flash frozen in liquid nitrogen. Samples were stored at –80°C prior to mass spectrometry analysis.

### Metabolite mass spectrometry analysis.

Two mouse retinas per sample were homogenized in 700 μL of MeOH 80% (v:v) solution while frozen in 13 × 100 borosilicate glass tubes using a mechanical homogenizer. We added 20 μL of 6 μM aqueous solution of 7-methyluric acid (2,4,5,6-13C4, 99%; 1,3,9-15N3, 98%) as internal standard (Cambridge Isotope Laboratories). Samples were thoroughly vortexed and centrifuged in a benchtop bucket centrifuge at 2500*g* for 10 minutes. Supernatant was transferred to a 2.0 mL microcentrifuge tube and the protein pellet was reextracted and supernatants were combined. The cell pellet was reserved for total soluble protein determination (Pierce BCA Protein Assay Kit, Thermo Fisher Scientific). Methanolic extracts were dried in a centrifugal evaporator. Dry extracts were reconstituted in 200 μL of water 0.1% formic acid (v:v). Sample (1 μL) was injected in the liquid chromotography–tandem mass spectrometry (LC-MS/MS) system for analysis. TCA metabolites were quantified using the mass spectrometric parameters and separations conditions described in the Shimadzu LC-MS/MS method package for cell culture profiling on a Nexera X2 UHPLC coupled to an LCMS-8060 (Shimadzu Scientific Instruments). LabSolutions V 5.82, LabSolutions V. 5.99 SP2, LabSolutions Insight V 2.0,and LabSolutions Insight Explore V3.7 SP3 program packages were used for data processing (Shimadzu Scientific Instruments). Supplemental Figure 5 shows results from this quantitative mass spectrometry for precise TCA cycle metabolites.

An expanded TCA metabolites panel — containing glutamine, glutamic acid, α-KG acid, and aspartic acid — was analyzed using alternative sample preparation and derivatization methodologies. Samples containing 2 mouse retinas were homogenized and extracted as described in the Shimadzu LC-MS/MS Method Package for Primary Metabolites (version 2.0, Shimadzu Scientific instruments; ref. 36). The final extract was split in 2 fractions and dried in a centrifugal evaporator. One of the fractions was reconstituted in 100 μL of HPLC water and 1 μL was injected in the LC-MS/MS system for analysis. The analysis was performed using the chromatographic conditions and instrument parameters implemented in the Shimadzu LC-MS/MS Method Package for Primary Metabolites on a Nexera X2 UHPLC coupled to an LCMS-8060 (Shimadzu Scientific Instruments). The second fractions were derivatized with 3-nitrophenylhydrazine to increase the sensitivity for α-ketoglutaric acid (37, 38), and 3 μL were injected in the LC-MS/MS system for analysis. The analytical system consisted of a Nexera X2 UHPLC coupled to an LCMS-8060 (Shimadzu Scientific Instruments). Chromatographic separation was achieved on a Titan C18 1.9 μm 100 × 2.1 mM (Supelco, Bellefonte) using a gradient elution with H2O 0.1% formic acid and MeOH:MeCN 1:1 (v:v) 0.1% formic acid.

Metabolite data were analyzed following previously published methods (39–41). Briefly, samples were normalized to total soluble protein, followed by calculating the percent difference between the individual sample and the mean value for the WT control group (the positive controls) from each mass spectrometry analysis. Data are presented as the percentage of WT concentration for each metabolite.

### Electroretinography.

Mice were dark-adapted overnight, manipulations were conducted under dim red light illumination, and recordings were made using the Phoenix MICRON Ganzfeld ERG System (Phoenix Technology Group). Pupils were dilated using topical 2.5% phenylephrine hydrochloride (Akorn Inc.) and 1% tropicamide (Sandoz). Mice were anesthetized by i.p. injection of 0.1 mL/10 g body weight of anesthesia (1 mL ketamine 100 mg/mL, Ketaset III, and 0.1 mL xylazine 20 mg/mL, Akorn Inc.) in 8.9 mL 1x PBS). Body temperature was maintained at 37°C during the procedure. Retinal responses were recorded at 4 different light intensity settings: –1.7, –1.1, 1.9, and 2.5 log cd•s/m^2^. The –1.1 log cd•s/m^2^ flash intensity setting was used for analysis of the dim light scotopic ERG response, and the 2.5 log cd•s/m^2^ was used for analysis of the bright light scotopic ERG response. Responses were taken from the Phoenix ERG readout in microvolts (μV) and a minimum of 7 measurements were recorded and averaged for each light setting. Photopic ERG was performed in a similar manner, but with a light adaptation period of 10 minutes at a setting of 4.6 log(cd/m^2^) preceding the recording. A minimum of 15 recordings at 3 different light intensity settings: 1.9, 2.5, and 3.1 log cd•s/m^2^ were analyzed. When photopic ERGs are recorded, there can be an artifact that appears as depolarization right at onset of the light stimulus. This was corrected for in the calculation of the amplitude of the photopic response by calculating the peak amplitude from the baseline of 0 μV. Data comparing 2 groups were analyzed via 2-tailed *t* test. Data comparing 2 groups over multiple time points were analyzed using multiple 2-tailed *t* tests with the Holm-Sidak method to correct for multiple comparisons.

### Histology and ONL quantification.

Mice were sacrificed following institutional guidelines, and the eyes enucleated. Enucleation was performed by proptosing the eye and placing a curved pair of forceps behind the eye, near the optic nerve,and gently pulling outward, releasing the eye and a portion of the optic nerve. Eyes were fixed at room temperature and embedded in paraffin, sectioned, and stained with H&E by Excalibur Pathology. Sectioning proceeded along the long axis of the segment, so that each section contained upper and lower retina as well as the posterior pole. Retinal sections were imaged using light microscopy (Zeiss Axio Observer, Carl Zeiss AG). Quantification of photoreceptor nuclei was conducted on several sections of the eye that contained the optic nerve, as follows: the distance between the optic nerve and the ciliary body was divided into 3, approximately equal, regions on each side of the eye. The number of nuclei in 4 columns was counted within each single region for the ONL of the retina. These counts were then used to determine the average thickness of the ONL for each individual animal and within each region of the retina, spanning from the ciliary body to the optic nerve head and back out to the ciliary body. For highly degenerated retinas (i.e., the 1-month-old *Rho^P23H/P23H^* mouse eyes), quantification was performed as described and the total ONL nuclei thickness was averaged for the entire retina. Statistical analysis was performed on the average ONL thickness for each retinal region and compared with other treatment or control groups in that same retinal region using multiple 2-tailed *t* tests with the Holm-Sidak method to correct for multiple comparisons.

### IHC.

Enucleated mouse eyes were fixed for 1 hour at room temperature in 4% paraformaldehyde. Cryopreservation was performed by sequential equilibration in increasing concentrations of 10%, 20%, and 30% sucrose solutions, each for 1 hour at room temperature. After an overnight equilibration at 4°C in 30% sucrose, the eyes were embedded in OCT media and frozen at –80°C. The OCT-embedded mouse eyes were sectioned in 10-μm thick slices and prepared on superfrost plus slides. Slides were washed with 1x PBS for 15 minutes to remove excess OCT. To ensure tissue permeability, slides were incubated in 0.2% Triton-X (catalog X100-100 ML, MilliporeSigma) solution for 15 minutes. Blocking was performed by incubating with blocking buffer (10% goat serum and 0.1% Triton-X-100 in PBS) for 2 hours at room temperature. The blocked tissues were then incubated overnight with appropriate primary antibody (GFAP, 1:250, catalog Mab360, MilliporeSigma; S-arrestin, 1:250, catalog PA1-731, Thermo Fisher Scientific; Red/Green Opsin, 1:250, catalog AB5405, MilliporeSigma; Rhodopsin, 1:250, catalog MABN15, MilliporeSigma) diluted in the blocking buffer. The next day, the tissues were equilibrated to room temperature and washed 3 times with 1X PBS and then incubated for 2 hours with appropriate Alexa Fluor secondary antibodies (Goat anti-Mouse Alexa Fluor 488, 1:500, catalog A11001 and Goat anti-Rabbit Alexa Fluor 594, 1:500, catalog A11012, Thermo Fisher Scientific). After washing 3 times in 1x PBS, sections were mounted with DAPI-mounting solution (catalog P36931, Thermo Fisher Scientific).

### Statistics.

Data are reported as mean ± SEM. GraphPad Prism Software (version 9.0) was used to generate graphs and perform statistical analysis unless otherwise indicated. Data comparing 2 groups were analyzed via 2-tailed *t* test. Data comparing more than 2 groups were analyzed using a 1-way ANOVA followed by Tukey’s post hoc multiple comparisons test. Data comparing 2 groups over multiple time points were analyzed using multiple 2-tailed *t* tests with the Holm-Sidak method to correct for multiple comparisons. Data with 2 independent variables were analyzed using 2-way ANOVA followed by Tukey’s post hoc multiple comparisons test (see individual sections in Methods). A *P* value of less than 0.05 was considered significant. Measurements were done blinded to experimental groups.

### Study approval.

All experiments were performed in accordance with the ARVO Statement for the Use of Animals in Ophthalmic and Visual Research and were all approved by the Animal Care and Use Committee at UT Southwestern Medical Center.

## Author contributions

KJW had full access to all the data in the study and takes responsibility for the integrity of the data and the accuracy of the data analysis. KJW conceived and designed the study. AAR, PDP, RG, and KJW acquired the data. AAR, PDP, RG, and KJW analyzed and interpreted the data. AAR, PDP, and KJW drafted the manuscript. AAR and KJW provided statistical analysis. KJW obtained funding. KJW provided administrative, technical, and material support. KJW supervised the study.

## Supplementary Material

Supplemental data

## Version 1 07/22/2021

In-Press Preview

## Version 2 09/08/2021

Electronic publication

## Version 3 09/08/2021

Updated graphical abstract
