# Supplementary material for: Replenishment of TCA cycle intermediates provides photoreceptor resilience against neurodegeneration during progression of retinitis pigmentosa
Source: JCI Insight. 2021 Sep 8;6(17):e150898. doi: 10.1172/jci.insight.150898 (PMC8492344; doi:10.1172/jci.insight.150898)
Supplement: Supplemental data [file jciinsight-6-150898-s342.pdf]

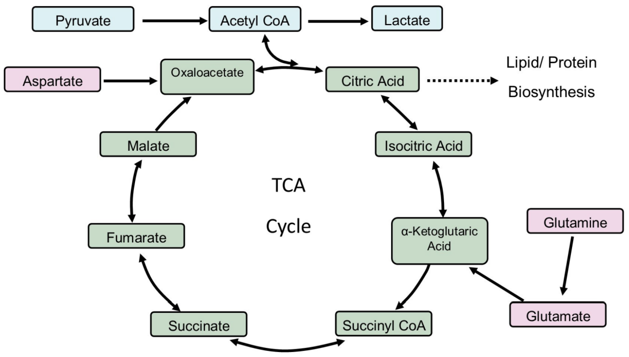

Supplementary Figure 1: Schematic of the tricarboxylic acid (TCA) cycle. Graphical representation of the TCA cycle and metabolites used for supplementation and profiling.

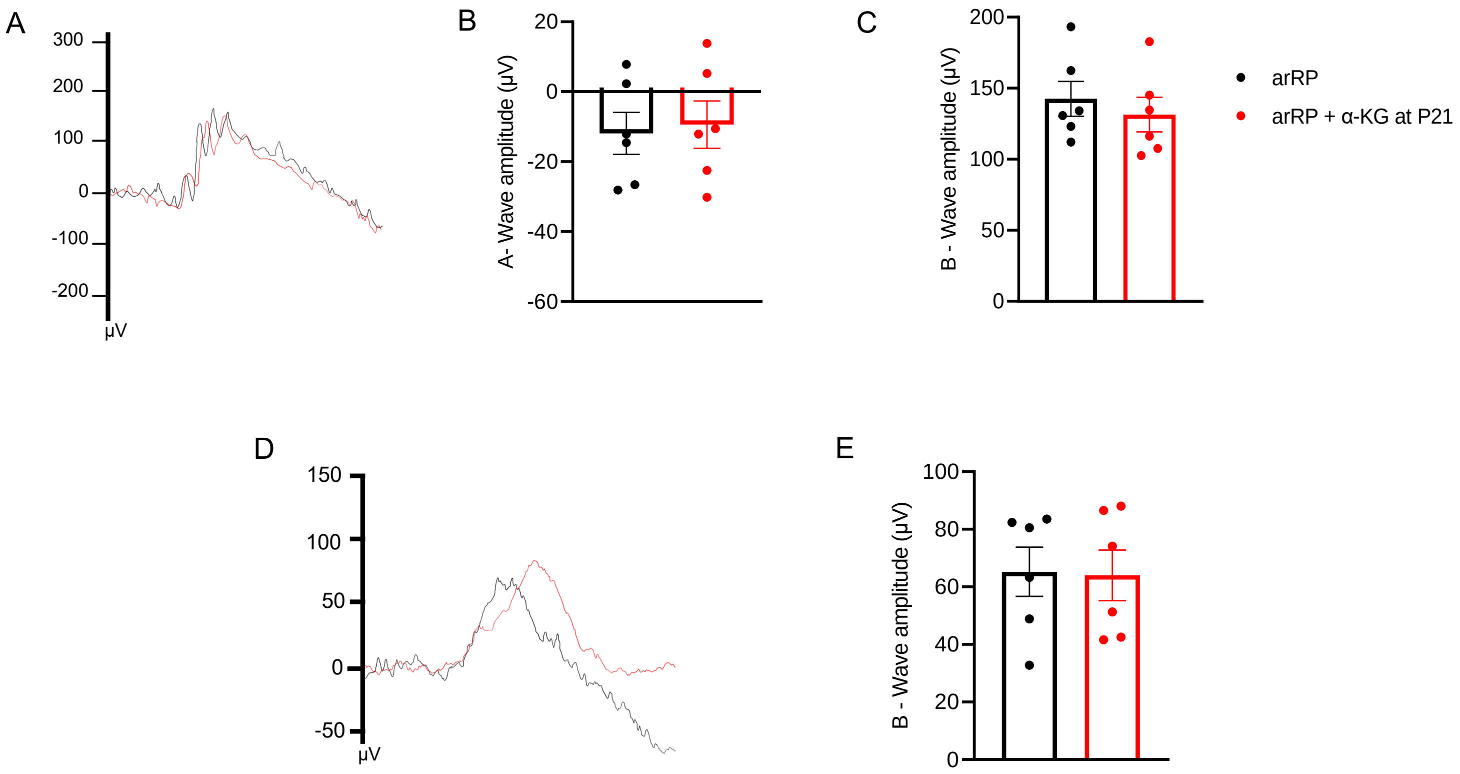

Supplementary Figure 2: Supplementation of α-KG at mid stage of disease has no detectable effect on visual response in the autosomal recessive retinitis pigmentosa (arRP) preclinical mouse model. (A) Representative average electroretinogram (ERG) traces for an untreated arRP mouse (black) and an arRP mouse treated with α-KG (red) beginning at post natal day (P)0, recorded at a 2.5 log cd•s/m^2 flash intensity. **(B)** Quantification of the bright light scotopic ERG visual response at one month of age, showing no statistical difference between treated and untreated groups in both the a-wave **(C)** and b-wave ERG response. **(D)** Representative average ERG traces at a -1.1 log cd•s/m^2 flash intensity. **(E)** Quantification of the dim light b-wave visual response. N= 6 eyes for both groups. Student’s t-test. Error bars = SEM.

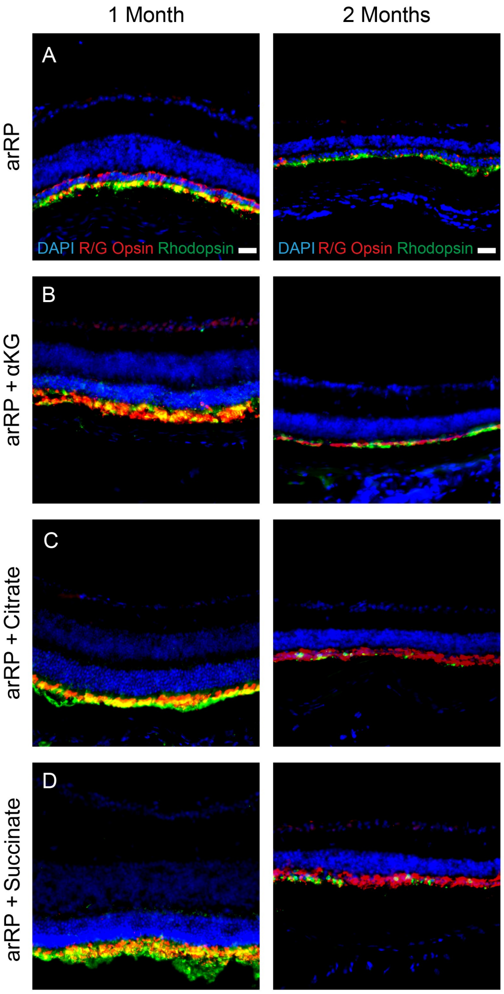

Supplementary Figure 3. Preservation of the cone photoreceptors in the retinas of a metabolite treated preclinical model of autosomal recessive retinitis pigmentosa (arRP). Retinal sections were stained for rhodopsin (rod photoreceptors) and red/green opsin (R/G Opsin; cone photoreceptors) at one and two months of age for **(A)** untreated arRP mice, **(B)** α-KG treated arRP mice, **(C)** citrate treated arRP mice, and **(D)** succinate treated arRP mice. Blue, DAPI (nuclei). Scale bar = 100 µm.

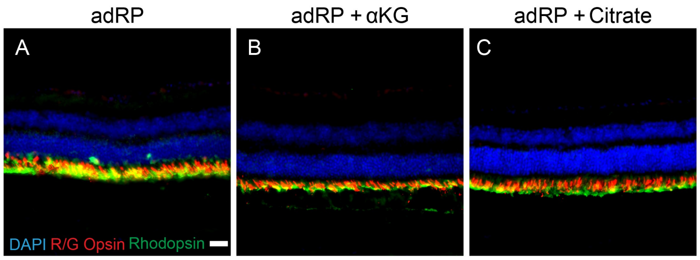

Supplementary Figure 4. Preservation of the cone photoreceptors in the retinas of a metabolite treated preclinical model of autosomal dominant retinitis pigmentosa (adRP). Retinal sections were stained for rhodopsin (rod photoreceptors) and red/green opsin (R/G Opsin; cone photoreceptors) at four months of age for **(A)** untreated adRP mice, **(B)** α-KG treated adRP mice, and **(C)** citrate treated adRP mice. Blue, DAPI (nuclei). Scale bar = 100 μm.

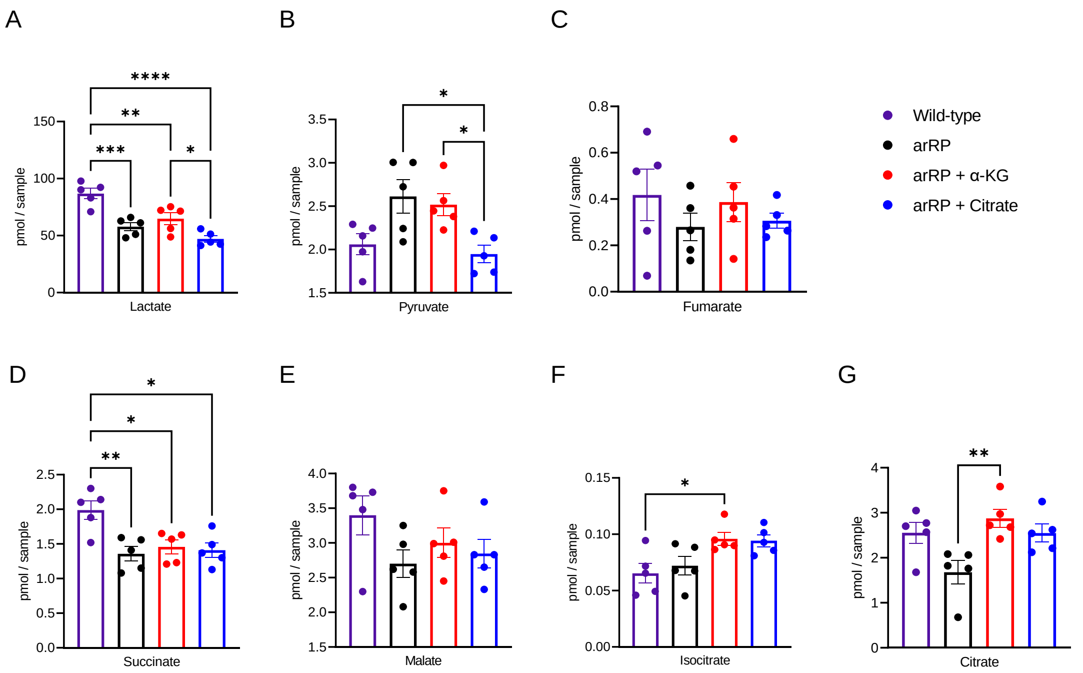

Supplementary Figure 5: Quantitative metabolite analysis in the autosomal recessive retinitis pigmentosa (arRP) preclinical mouse model. Mass spectrometry was used to profile metabolites in the neural retinas of wild-type (purple), arRP (black), arRP treated with α-KG (red), and arRP treated with citrate (blue) at one month of age for **(A)** lactate, **(B)** pyruvate, **(C)** fumarate, **(D)** succinate, **(E)** malate, **(F)** isocitrate and **(G)** citrate. Samples were normalized to total soluble protein and displayed as pmol per sample of two retinas. N = 10 retinas. One-way ANOVA with Tukey's multiple comparison's correction. Error bars = SEM. *, $p < 0.05$; **, $p < 0.01$; ***, $p < 0.001$; ****, $p < 0.0001$.
